# Thank You for NOT Smoking

**Published:** 2014-05-01

**Authors:** Pamela Hallquist Viale

The Surgeon General’s report on smoking was just released, coinciding with the 50th anniversary of the original report issued in 1964. The report, entitled "The Health Consequences of Smoking—50 Years of Progress," is alarming. Although awareness of the dangers of smoking has indeed increased, the cost of smoking continues to be shockingly high, perhaps higher than we originally calculated.

## Physical Effects

In the United States, smoking-related disease remains one of the most significant public health calamities. Although we have made great progress in tobacco control, it isn’t enough. Since the first Surgeon General’s report in 1964, more than 20 million Americans have died as a result of smoking. Most of those individuals were smokers, but almost 2.5 million of them were nonsmokers felled by heart disease or lung cancer resulting from second-hand smoke (US Department of Health and Human Services [HHS], 2014).

Cigarette smoking is killing more Americans per year than previously thought (almost 480,000, up from 443,000) and is a cause of many other cancers, including liver and colorectal cancers. Smoking affects every organ of the body and is now linked to other conditions such as rheumatoid arthritis. It is newly connected to chronic diseases such as macular degeneration and diabetes, in addition to the multitude of health consequences previously known. Exposure to second-hand smoke can be a cause of stroke and impaired immune function.

The number of smokers has declined, but the risk for smoking-related disease and death has not; today’s smokers have a significantly higher risk for lung cancer and chronic obstructive pulmonary disease compared with smokers in 1964, even when smoking fewer cigarettes (HHS, 2014). Ten times as many Americans have died prematurely from cigarette smoking when compared with those who have died in all of the wars fought by the United States in our entire history. Gender can no longer afford any protection: For the first time, women have been found to have the same high risk of death from lung cancer as men. In addition, the risk of dying from coronary heart disease among women 35 and older is now higher than the risk for men (HHS, 2014).

## Tremendous Costs

The tragic statistics translate to a huge yet avoidable public health menace. The US annual costs attributed to smoking range between $289 billion and $333 billion. This figure includes $130 billion for necessary medical support; lost productivity due to premature death and death caused by second-hand smoke are also a part of that estimate (HHS, 2014). Although the US Food and Drug Administration (FDA) has continued to battle the tobacco industry regarding direct advertising of its products, the industry continues to spend $35 million dollars daily on marketing (Layton, 2010). Despite the fact that the last television commercial endorsing smoking was shown in 1971, smoking print advertisements continue to exist.

Tragically, middle and high school students are becoming smokers; the tobacco industry aims targeted advertisements and activities toward this malleable group to get them smoking young. After that, addiction to nicotine helps keep them life-long smokers. The Surgeon General’s report also discusses the significant disparities in tobacco use among different racial and ethnic groups (HHS, 2014).

## What Can Be Done?

The Surgeon General’s report should be mandatory reading for all health-care professionals and members of the lay public. Although the statistics regarding the many dangers of smoking may already be known, I think the conclusion of the report still needs to be emphasized. The evidence detailed in the Surgeon General’s report demonstrates that the decline in tobacco use in the near future will not be sufficient. Ending the avoidable health consequences of smoking and premature death will not occur quickly enough unless additional action is taken.

The Surgeon General’s report calls for specific strategies to combat the effects of smoking. These interventions include creating media campaigns to counteract industry marketing, raising excise cigarette taxes significantly, allowing the Affordable Care Act to provide barrier-free tobacco cessation treatment, expanding smoking cessation program efforts in primary and specialty care settings, supporting FDA-affected tobacco product regulation, funding statewide tobacco control programs, and extending comprehensive smoke-free indoor protection to 100% of the US population (HHS, 2014).

## Personal Reflections

The Surgeon General’s report stops short of recommending that smoking products be made illegal. Although the idea of banning cigarettes and other tobacco products is one that pleases me on so many levels, realistically I can’t be sure that that move wouldn’t inspire a culture similar to the ones marijuana smokers face today.

However, I do have a vested interest in the complete cessation of smoking. Smoking contributed directly to the death of my mother, who passed away from small cell lung cancer at the relatively young age of 62. Smoking killed her twin sister at age 77, who died of adenocarcinoma of the lung. Smoking killed my mother’s brother, who died of metastatic lung cancer at the age of 58. Smoking indirectly led to my grandmother’s death when she, a lifelong smoker, died of respiratory failure at age 66. I can’t find anything to recommend about smoking. Clearly, our efforts must be increased in combating this scourge.

## APSHO at JADPRO Live

It’s time to save the date for our next live educational meeting, APSHO at JADPRO Live, to be held October 30 through November 2, 2014, in Orlando, Florida. We promise you 3 days of focused education aimed at the advanced practitioner. There will be multiple opportunities for you to join APSHO committees and participate in the evolution of this newly formed organization devoted to your educational and supportive needs! Visit www.apsho.org for more information on the Society and keep an eye on www.advancedpractitioner.com for updates on our next conference! We hope to see you in Orlando this fall!

## References

[1] L. Layton (2010). New FDA rules will greatly restrict tobacco advertising and sales.. *The Washington Post.*.

[2] (2014). The health consequences of smoking—50 years of progress: A report of the Surgeon General.. *US Department of Health and Human Services.*.

